# Are current guidelines for categorization of visual impairment in India appropriate?

**DOI:** 10.4103/0301-4738.57144

**Published:** 2009

**Authors:** Parveen K Monga, Binay P Parwal, Jolly Rohatgi, Upreet Dhaliwal

**Affiliations:** 1 Jindal Institute of Medical Sciences, Hisar, India; 2 Department of Medicine, University College of Medical Sciences and Guru Teg Bahadur Hospital, Delhi, India; 3 Department of Ophthalmology, University College of Medical Sciences and Guru Teg Bahadur Hospital, Delhi, India

**Keywords:** Blindness, disability evaluation, low, vision, visual acuity

## Abstract

**Context:**

Visual disability in India is categorized based on severity. Sometimes the disabled person does not fit unambiguously into any of the categories.

**Aims:**

To identify and quantify disability that does not fit in the current classification, and propose a new classification that includes all levels of vision.

**Settings and Design:**

Retrospective chart review of visual disability awarded in a teaching hospital.

**Materials and Methods:**

The last hundred records of patients who had been classified as visually disabled were screened for vision in both eyes and percentage disability awarded. Data were handled in accordance with the Helsinki Declaration.

**Results:**

Twenty-one patients had been classified as having 30% disability, seven each had 40% and 75%, and 65 had 100% disability. Eleven of them did not fall into any of the current categories, forcing the disability board to use its own judgment. There was a tendency to over-grade the disability (seven of 11; 63.6%). The classification proposed by us is based on the national program for control of blindness' definition of normal vision (20/20 to 20/60), low vision (<20/60 to 20/200), economic blindness (<20/200 to 20/400) and social blindness (<20/400). It ranges from the mildest disability (normal vision in one eye, low vision in the other) up to the most severe grade (social blindness in both eyes).

**Conclusions:**

The current classification of visual disabilities does not include all combinations of vision; some disabled patients cannot be categorized. The classification proposed by us is comprehensive, progresses logically, and follows the definitions of the national program.

Visual impairment disability in India is categorized based on its severity. Percentages are accorded as proposed by a subcommittee constituted by the Ministry of Social Justice and Empowerment in 1999. The categories of visual disability are notified in the Gazette of India, extraordinary, 2001 and are followed all over the country.[1] On using the classification over the years, we have come across instances of visual disability that do not fit into any of the categories. Thus, disability certification board members have to rely upon their own discretion in categorizing some cases. This study was aimed to identify and quantify those cases and propose a new classification that includes all levels of visual acuity.

## Materials and Methods

The current classification of visual disability was critically examined to determine which combinations of vision did not find a place in the classification. In addition, the last hundred records of patients who had been classified as visually disabled in this hospital were screened. Data relating to vision in both eyes and the disability percentage accorded by the disability board was retrieved and entered into an MS Excel worksheet. To protect anonymity, the names of patients were not noted; we identified them by serial numbers. Data were handled in accordance with the ethical standards of the Helsinki Declaration of 1975, as revised in 2000. The disability percentage recorded by the board was compared with the current classification, in order to determine the extent and type of disparity, if any. Finally, a classification was created using the national program for control of blindness (NPCB) definitions of normal vision, low vision, and blindness, and including every possible combination of vision in the two eyes of a visually disabled patient.

## Results

Table 1 shows the categories of visual disability that are currently in use all over India.[1] Of the 100 visual handicap records that were screened for this study, 21 persons had been classified as having 30% disability, seven each had 40% and 75%, and 65 had 100% visual disability. Eleven of these persons did not fall into any of the current categories of visual disability [Table 2]; nevertheless, the institutional visual disability board had used its own judgment. The percentage of disability awarded to these 11 persons is detailed in Table 2. The tendency was to over-grade the disability (seven of 11 disabilities; 64%), giving the applicant the benefit of this loophole in the classification system.

**Table 1:** Categories of visual disability (classification currently in use)^1^

Category	Better eye*	Worse eye*	Percentage impairment
Category 0	20/30-20/60	20/80 to 20/120	20
Category I	20/60-20/120	20/200 to Nil	40
Category II	20/130-20/300 or field of vision 10°-20°	20/400 to Nil	75
Category III	20/400 to 20/1200 or field of vision 10°	20/8000 to Nil	100
Category IV	20/8000 to Nil or field of vision 10°	20/8000 to Nil	100
One-eyed persons	20/20	20/8000 to Nil or field of vision 10°	30

* with correcting lenses

**Table 2:** Vision grades that do not fit into the current visual disability classification

No place in the current disability classification		Number of patients in our series (n=100)	Disability awarded by Board members (%)	Interpretation of disability awarded*
Vision in the better eye	Vision in the worse eye			
20/20	20/80 to 20/1200	1	30	Over-graded†
20/30	20/200 to no PL	3	30	Under-graded†
20/40	20/200 to no PL	1	40	Over-graded
20/80	20/80 to 20/120	0	-	-
20/120	20/120	1	40	Over-graded
20/200	20/200 to 20/300	1	75	Over-graded
20/240	20/240 to 20/300	0	-	-
20/300	20/300	0	-	-
20/400	20/400 to 20/1200	1	75	Under-graded
		1	100	Over-graded†
20/600	20/600 to 20/1200	0	-	-
20/1200	20/1200	2	100	Over-graded†
Total		11		

PL-perception of light

* based on the existing classification of visual disability^1^

† close to appropriate

## Discussion

Most persons seeking visual disability certification in this institution do so for severe disability (75-100% category). However, even one-eyed persons (30% disability) request certification. This occurs in spite of the Ministry of Health's notification that a person with <40% disability will not be eligible for benefits/concessions.[1] They contend that clubbing one-eyed persons with other visual handicap categories will dilute the benefit to the latter. In this respect, certification of one-eyed persons might be considered a wasteful exercise, causing unnecessary expenditure of resources in the certification process. On the other hand, in multiple disabilities, even a relatively small visual disability could make a difference in the final certification. There is a provision to combine the effects of multiple disabilities, particularly those involving neurological and musculoskeletal systems.[1] The disability with the lower score (b) is added to the highest score (a) and final disability is calculated using the formula:

Combined disability = a+[b(100−a)/100]

This means that a person with 25% neurological and 20% visual disability would have a combined disability of 40%, thus entitling him to benefits and concessions.

The new classification suggested by us [Table 3] includes all combinations of vision in the two eyes. There are nine possible grades of disability based on the NPCB classification of normal vision (20/20 to 20/60), low vision (<20/60 to 20/200), economic blindness (<20/200 to 20/400) and social blindness (<20/400).[2] However, while the NPCB definitions use presenting vision, we recommend best-corrected visual acuity (BCVA). Assessing presenting vision is practicable in field situations, but when a person presents for disability certification, every attempt should be made to improve vision before certifying. The current classification of disability too uses BCVA.[1]

**Table 3:** Proposed new visual disability classification based on the NPCB definitions of normal vision, low vision, economic blindness and social blindness

BCVA in the better eye	BCVA in the worse eye	Percentage of disability
Normal vision	Normal vision	None
20/20 to 20/60	20/20 to 20/60	
Normal vision	Low vision	10
20/20 to 20/60	<20/60 to 20/200	
Normal vision	Economic blindness*	20
20/20 to 20/60	<20/200 to 20/400	
Normal vision	Social blindness†	30
20/20 to 20/60	<20/400	
Low vision	Low vision	40
<20/60 to 20/200	<20/60 to 20/200	
Low vision	Economic blindness*	50
<20/60 to 20/200	<20/200 to 20/400	
Low vision	Social blindness†	60
<20/60 to 20/200	<20/400	
Economic blindness*	Economic blindness*	70
<20/200 to 20/400	<20/200 to 20/400	
Economic blindness*	Social blindness†	80
<20/200 to 20/400	<20/400	
Social blindness†	Social blindness†	90
<20/400	<20/400	
No perception of light	No perception of light	100

BCVA-best-corrected visual acuity

* or field of vision greater than 10 but no more than 20 degrees

† Or field of vision ≤10 degrees

The proposed classification is comparable with the classification of visual impairment recommended by the World Health Organization (WHO) [Table 4].[3] Persons with low vision in both eyes, in the proposed classification, correspond to category I of the WHO classification, persons with bilateral economic blindness correspond to category II, and persons with bilateral social blindness correspond to categories III, IV and V. As in the WHO classification, persons with no perception of light have been given a separate identity. These persons are likely to have incurable ocular conditions and be in need of long-term support and concessions. The proposed classification is in agreement with the WHO classification for fields of ten degrees or less, and closely comparable with the NPCB classification for fields greater than ten degrees and up to 20 degrees.

**Table 4:** Classification of severity of visual impairment recommended by WHO^3^

Category of visual impairment	Visual acuity with both eyes using best possible correction*	
	
	Maximum less than	Minimum equal to or better than
	6/18	6/60
1	3/10 (0.3)	1/10 (0.1)
	20/70	20/200
	6/60	3/60
2	1/10 (0.1)	1/20 (0.05)
	20/200	20/400
	3/60	1/60 (finger counting at 1 meter)
3	1/20 (0.05)	1/50 (0.02)
	20/400	5/300 (20/1200)
	1/60 (finger counting at 1 meter)	
4	1/50 (0.02)5/300	Light perception
5	No light perception	
9	Undetermined or unspecified	

a: If the extent of the visual field is to be considered also, patients with a field of less than 10 but more than 5 degrees around central fixation should be placed in category 3 and patients with a field less than 5 degrees around central fixation should be placed in category 4, even if the central acuity is not impaired.

b: These categories are intended to correspond with the fourth digit of the numbering system used in the International Classification Diseases. In this system, the digit 9 customarily signifies “unspecified”.

The proposed classification is comparable with the one currently in use in that vision reduction upto and including 20/60, in both eyes, is considered normal. The current classification awards 20, 30 or 40% disability to persons with normal vision in one eye but fails to categorize many visual combinations [Table 2]. Forty percent disability will enable one-eyed persons to compete for benefits and concessions with the severely visually disabled, to the disadvantage of the latter. The proposed classification has also created three classes for one-eyed persons, but does not go higher than 30% disability.

Most combinations for persons with bilateral low vision are missing from the current classification. The rest are awarded 40% disability. The proposed classification misses none, awarding 40% disability to all. Persons with low vision in the better eye and economic blindness in the worse eye are awarded 40% disability in the current classification; some visual combinations are missing. The proposed classification misses none, and awards them 50% disability. The difference of 10% in disability status should not make much difference since both will be eligible for concessions or benefits.

For persons with low vision in the better eye and social blindness in the worse eye, the current classification awards 40% or 75% disability. In our opinion, 40% disability is unfair since these persons are definitely more disadvantaged than those described in the preceding paragraph (low vision in one eye and economic blindness in the other). On the other hand, 75% disability may be too generous; they are in a better position than persons with economic blindness in both eyes. Thus, the proposed classification awards them 60% disability.

Persons with economic blindness in both eyes are awarded 75% disability using the current, and 70% using the proposed classification. Many visual combinations are missing in the current classification but not in the proposed one. The current classification awards 75 or 100% disability to persons with economic blindness in the better eye and social blindness in the worse eye, but misses some visual combinations. The proposed classification misses none and awards 80% disability to them.

The current classification awards 100% disability to persons who have social blindness in both eyes, but misses many visual combinations. We suggest such persons be awarded 90% disability except when they have no perception of light in both eyes (suggesting an incurable condition), when they can be awarded 100% disability.

It was considered during formulation of this classification, that visual disability of <40% could be abolished altogether since no benefits or concessions accrue to them. However, if multiple disabilities are present, even 20% visual disability may allow the person to get benefit from educational and job schemes. Thus, lower degrees of visual disability must continue to have a place in the disability classification. In the proposed classification, the difference between grades is 10%. The spectrum varies from the mildest disability (normal BCVA in one eye but low vision in the other) up to the most severe grade (social blindness in both eyes).

The proposed classification has several strengths to recommend it. It follows the NPCB definitions of low vision and blindness, thus being in uniformity with the national program. It is in tandem with the WHO classification of visual disability, thus giving it international comparability. It is more or less comparable with the current classification in India; the field defects commensurate with low vision and blindness are also the same; BCVA is the criterion in both classifications. It includes every possible combination of vision in the two eyes. In addition, it provides a wider range of disability. This may be of use to a person having multiple disabilities. The proposed categories follow a natural progression making them logical and easy to remember.
